# Awareness of CPR-induced consciousness by UK paramedics

**DOI:** 10.29045/14784726.2019.06.4.1.1

**Published:** 2019-06-01

**Authors:** Ben Mays, Pete Gregory, Ceri Sudron, Tim Kilner

**Affiliations:** Yorkshire Ambulance Service NHS Trust: ORCID iD: https://orcid.org/0000-0002-7129-9885; University of Wolverhampton: ORCID iD: https://orcid.org/0000-0001-9845-0920; University of Wolverhampton: ORCID iD: https://orcid.org/0000-0003-0211-0628; University of Worcester: ORCID iD: https://orcid.org/0000-0001-7725-4402

**Keywords:** cardiac arrest, cardiopulmonary resuscitation, consciousness, paramedic

## Abstract

**Objectives:**

Guidelines for the management of hospital cardiac arrest advocate minimally interrupted chest compressions in order to maintain cerebral perfusion pressures and improve the likelihood of a positive outcome. One condition that may lead to interruptions in the delivery of chest compressions is cardiopulmonary resuscitation induced consciousness (CPR-IC). This study investigates UK paramedics’ understanding of CPR-IC and how they came by their knowledge.

**Methods:**

This study was a cross-sectional survey of paramedics who were registered with the Health and Care Professions Council (HCPC) and practising in the United Kingdom at the time of the survey. Participants completed an online survey; the first two sections are reported here. Section 1 asked for demographic data pertinent to the study outcomes and section 2 asked participants to explain what they understood about CPR-IC and the source of their information.

**Results:**

A total of 293 eligible participants completed the survey. Most had over 5 years’ experience as a paramedic and declared no specialist clinical role. Over 50% of respondents said that they had heard of CPR-IC prior to the study and the majority of those provided an explanation that demonstrated some understanding when compared with the definition used by the study team. Over 40% of respondents became aware of CPR-IC after having witnessed it in clinical practice.

**Conclusion:**

Nearly half of the study participants were not aware of CPR-IC, and few have had formal training on the phenomenon. There is a clear need for further education on CPR-IC in order for paramedics to better manage it when presented with it in practice.

## Introduction

Guidelines for the management of cardiac arrest strongly advocate the provision of high quality, minimally interrupted chest compressions (Soar, Deakin, Lockey, Nolan, & Perkins, 2015). A condition that may impact on the provision of high-quality compressions is a phenomenon known as cardiopulmonary resuscitation induced consciousness (CPR-IC), whereby a patient appears to regain some level of consciousness during cardiac arrest when chest compressions are being performed, even though they have no return of spontaneous circulation (ROSC).

Epidemiological data surrounding CPR-IC are scant, with only a single retrospective study conducted in Australia (Olaussen et al., 2017). Olaussen et al. (2017) found that of 16,558 out-of-hospital cardiac arrests (OHCA), 0.7% appeared to show signs of consciousness. Characteristics which increased the likelihood of CPR-IC included clinician witnessed cardiac arrest, short response times and a presenting rhythm of ventricular fibrillation or ventricular tachycardia.

While there are case reports in the published literature (Bihari & Rajajee, 2008; Lewinter, Carden, Nowak, Enriquez, & Martin, 1989; Tobin & Mihm, 2009), there is limited high quality research into this emerging topic. There is also a wide variation in the level of consciousness displayed in these cases, from patients who moved their limbs and groaned, to patients who displayed higher levels of cognitive function, for example following commands and making purposeful movements such as pushing clinicians away, attempting to remove devices (e.g. endotracheal tubes) and following instructions (Olaussen et al., 2015). There is no universally accepted definition of CPR-IC, although one group (Olaussen et al., 2016) has classified signs into non-interfering (eye-opening, agonal breaths or mild restlessness) and interfering (purposeful movement, withdrawing from CPR, attempting to pull out airway-securing devices, combating or pushing rescuer away) categories.

The aim of this study was to identify how many UK paramedics are aware of CPR-IC and how they came to learn about the topic.

## Methods

### Design and instrumentation

This study was a cross-sectional survey of paramedics who were registered with the Health and Care Professions Council (HCPC) and practising in the United Kingdom at the time of the survey. The survey was conducted using an online survey tool (https://www.onlinesurveys.ac.uk/) between 8 December 2017 and 17 January 2018, following the receipt of ethical approval. The decision to close the survey on 17 January was made because there had been no responses made in the preceding 5 days. The final survey (Supplementary 1) was produced from several pilot questionnaires that were tested within the Institute of Health at the University of Wolverhampton prior to release. In the absence of a formal definition of CPR-IC, we proposed our own definition as ‘signs of consciousness perceptible to the rescuer during the application of cardiopulmonary resuscitation in a patient in confirmed cardiac arrest’. Determination of cardiac arrest would need to be clinical judgement based upon the absence of a palpable pulse at a major site accompanied by an ECG rhythm check. It was understood that there may be a potential for a pseudo-pulseless electrical activity (PEA) to be mistaken for CPR-IC but that it would be difficult to remove this bias. This would enable us to determine the concordance of participants’ understanding with our own interpretation. Our definition of CPR-IC was based upon previous literature and derived from consensus within the study team, the wider paramedic teaching team and the professor of nursing at the University of Wolverhampton.

### Participants

The survey was promoted to paramedics via social media, the College of Paramedics and by word-of-mouth. Statutory ambulance services within the NHS had the option to distribute the survey but generally opted not to as despite university level ethics, ambulance services felt uncomfortable distributing research without NHS research ethics committee approval. Any paramedic who was registered and working within the United Kingdom at the time of the study was eligible to complete the survey, irrespective of their current employment. In the United Kingdom, the majority of paramedics are employed within NHS ambulance services but there is a growing number who work outside the NHS in a variety of emerging and established roles. It was important not to restrict the population to those employed by NHS ambulance services as it would potentially eliminate a large portion of the paramedic population. Responding to the survey was both voluntary and anonymous, with no individual identifying information being recorded.

### Data analysis

Numerical descriptive data were analysed using the online survey tool, while free text answers were exported and themes identified by two of the study team (BM and CS) working independently. Once the themes had been identified, PG reviewed the coding to assess for concordance between the two investigators. Any anomalies were discussed within the study group to achieve consensus. Finally, the study definition of CPR-IC was compared to the participants’ definitions.

### Results

**Figure 1:**
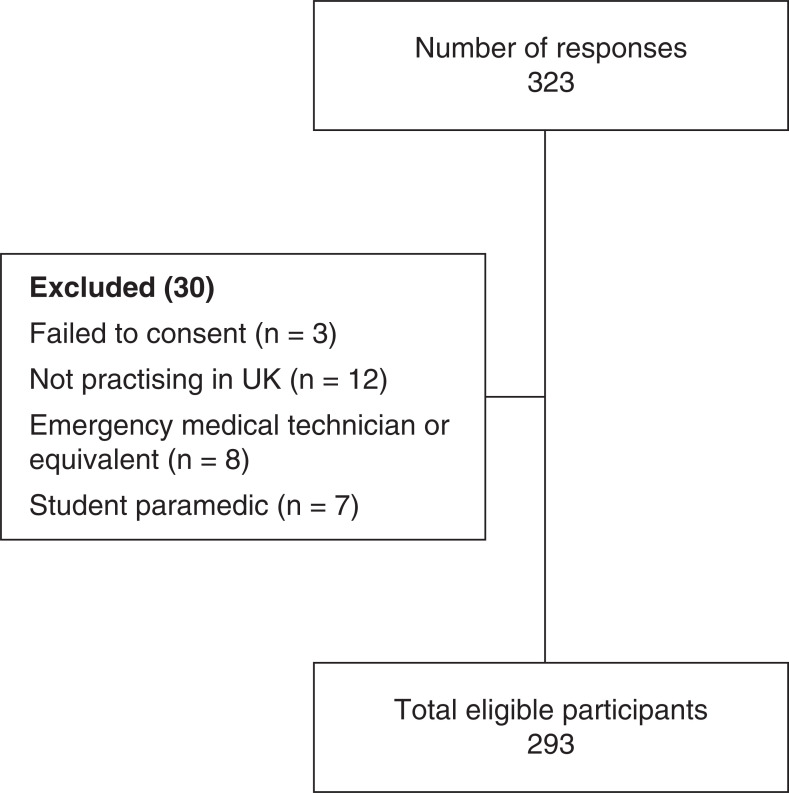
Figure 1. Study participants.

A total of 323 participants commenced the survey, of which 320 consented to participate in the study. The three who did not provide consent were not allowed to access the survey questions. Of the remaining 320 participants, 27 were excluded as they were ineligible to participate in the study (Figure 1). This left 293 participants, comprising approximately 1.17% of the total number of UK registered paramedics at the time (25,000).

### Participant demographics

The majority of respondents were currently working in clinical practice in a ‘generalist’ paramedic role, had qualified as a paramedic via a higher education route and had attended more than 10 cardiac arrests in which CPR was attempted during their career (Table 1).

**Table 1.** Summary of survey respondent demographic information.

	n (%)
**Currency of clinical practice**	
Current	271 (92.5)
Within the last 12 months	5 (1.7)
Between 1 and 5 years ago	8 (2.7)
More than 5 years ago	9 (3.1)
**Length of service (WTE)**	
0–4 years	93 (31.7)
5–9 years	93 (31.7)
10 years or more	107 (36.5)
**Education route**	
IHCD (or equivalent)	89 (30.4)
Certificate of Higher Education	10 (3.4)
Foundation degree/Diploma of Higher Education	132 (45.1)
BSc/BSc (Hons)	62 (21.1)
**Specialist role**	
No specialist role	191 (65.2)
Primary care	22 (7.5)
Critical care	39 (13.3)
Other	41 (14)
**Number of cardiac arrests attended**	
Up to 10	21 (7.2)
11–50	122 (41.6)
More than 50	150 (51.2)

Of the respondents, 157 (53.6%) indicated that they were familiar with the term CPR-IC prior to completing the survey and provided a response to the question that asked them to explain their understanding of the term. Of the respondents, 140 discussed some but not all of the components of CPR-IC and were deemed to show varying degrees of understanding of the term when compared with our definition. The most commonly cited themes are identified in Table 2. Examples of explanations that were deemed to show an understanding of CPR-IC included:

**Table 2.** Common themes when describing CPR-IC.

Correct themes	Number of times mentioned
Motor response/movement	33
Cerebral perfusion pressure	17
Glasgow Coma Scale score	15
Eye opening	13
High/good quality CPR	12
Verbalisation	10
Eyes, verbal and motor responses (specifically)	7

A state in cardiac arrest [in] which the standard of CPR is so high that the brain is being perfused in such a manor [sic] that the patient is ‘light’ and possibly aware of surroundings/pain/incident/emotions. (Participant 27928723)

Where consciousness occurs as a result of the effectiveness of CPR not because of physiological improvements. (Participant 27986012)

When effective CPR was performed to the point where the pt was able to achieve some level of consciousness. (Participant 27993915)

When asked how they became aware of the phenomenon (Table 3), they identified five key themes: formal teaching, informal learning, word-of-mouth, witnessing CPR-IC out-of-hospital and witnessing it in-hospital.

**Table 3.** Methods by which respondents became aware of CPR-IC.

Method of learning about CPR-IC	n (%)
Formal learning	41 (26.1)
Informal learning	22 (14.0)
Word-of-mouth	23 (14.6)
Witnessed CPR-IC out-of-hospital	63 (40.1)
Witnessed CPR-IC in-hospital	3 (2.0)
Did not respond	5 (3.2)
Total	157 (100.0)

Of the 17 who were deemed to have incorrectly defined CPR-IC, seven identified it as a ROSC, seven discussed awareness rather than consciousness and one discussed low flow PEA. One participant said that they had ‘no idea’ and one other inserted an ‘N’ in the text box. Notably, 31 participants said that they did not know what CPR-IC was prior to commencement of the study but declared that they had experienced the phenomenon on two or more occasions.

Formal learning included being taught through universities, training received through the Lucas™ device and reading of peer-reviewed academic journal articles. Informal learning was derived largely through blogs, podcasts and social media, with a number specifically mentioning Twitter. Those who learnt through word-of-mouth typically reported hearing from other crews who had experienced the phenomenon:

I heard stories about patients regaining consciousness when a Lucas was being used. (Participant 27998235)

Over 41% of respondents identified that they had learnt about CPR-IC when exposed to it in their practice. In what was not an uncommon reply, one respondent reports learning of CPR-IC for the first time:

When performing CPR on a patient [. . .] he was telling me to ‘get off’ and trying to pull my hands off his chest. When we stopped CPR he became unconscious. (Participant 28556665)

## Discussion

Despite the wealth of resuscitation-related studies, there is a paucity of research addressing CPR-IC. Given that CPR-IC could be considered an atypical (but not rare) phenomenon (Grandi et al., 2017), it is important that paramedics are aware of the potential for CPR-IC to occur and are able to correctly define and identify its characteristics. In our study, 136/293 (46.4%) had not heard of CPR-IC prior to completing the study and 17/293 (5.8%) who said that they had heard of it failed to offer an explanation that accorded with our proposed definition. This means that over half of our study population (153/293, 52.2%) had either no understanding or an incorrect understanding of the phenomenon. It is not clear whether this affected the management of patients who presented with CPR-IC but it is possible that this may have caused some confusion during the episode and impacted upon the continuance of resuscitation attempts.

The results also highlight that few respondents (41/152, 27%) had learnt about CPR-IC in formal teaching sessions, which means that fewer than 14% (41/293) of the overall study population had received formal teaching around CPR-IC. This concern is also reflected in the relatively high number of participants who first experienced the phenomenon when presented with it in their clinical practice. Cardiac arrest in the out-of-hospital environment can be stressful and it is reasonable to suggest that overall effectiveness in the management of cardiac arrest could be impaired where a previously unknown phenomenon is experienced. This would be even more likely if the patient displayed features consistent with interfering CPR-IC as described by Olaussen et al. (2016). In addition, there are further ethical considerations that may need to be explored when considering the management of a patient who has regained some level of consciousness due to the effects of CPR. These include whether survivors have any post-traumatic symptoms and whether there is a humanitarian requirement for sedation in such patients. It is recommended that discussion around CPR-IC be included in all pre-registration paramedic programmes and form part of mandatory training for registered paramedics.

### Limitations

The study was primarily limited by the method of distribution, as most NHS Trusts decided against promoting the study to their workforce due to lack of NHS research ethics committee approval (despite university level ethics). The study was promoted primarily through social media and word-of-mouth, which may mean that those who do not use social media were unaware of the study. The sample captured approximately 1.2% of the total number of registered paramedics (25,000) but it is unclear whether this sample was entirely representative of the wider paramedic population.

## Conclusion

Around 50% of our study population was aware of CPR-IC and able to accurately define it when compared with the definition – ‘Signs of consciousness perceptible to the rescuer during the application of cardiopulmonary resuscitation in a patient in confirmed cardiac arrest’. However, few had undertaken any formal learning on the topic and most respondents learnt of the phenomenon through attending patients displaying signs and symptoms of CPR-IC. Based on the results of this survey, there is a need for further education on CPR-IC in order for paramedics to prepare for the ethical and clinical dilemmas they may face when confronted with such patients.

## Conflict of interest

None declared.

## Ethics

Ethical approval was granted by the University of Wolverhampton’s ethics committee in November 2017.

## Funding

College of Paramedics, Small Research Grant.
